# Characteristics of psychiatric patients with hypokalemia after yokukansan administration: A retrospective study

**DOI:** 10.1002/pcn5.76

**Published:** 2023-02-01

**Authors:** Kazuya Yasuda, Ryuichiro Takeda, Ryuji Ikeda, Yasushi Ishida

**Affiliations:** ^1^ Department of Pharmacy University of Miyazaki Hospital Miyazaki Japan; ^2^ Health Care and Safety Center University of Miyazaki Miyazaki Japan; ^3^ Department of Psychiatry, Faculty of Medicine University of Miyazaki Miyazaki Japan

**Keywords:** dementia, hypokalemia, psychiatric disorders, risk factors, yokukansan

## Abstract

**Aim:**

Yokukansan is a Japanese herbal medicine used in psychiatry to treat behavioral and psychological symptoms of dementia and other psychiatric symptoms. However, the glycyrrhizic acid included in this medicine can cause pseudoaldosteronism and hypokalemia. We aimed to identify the risk factors for hypokalemia due to yokukansan.

**Methods:**

A retrospective cohort study was conducted on patients previously treated with yokukansan. The risk factors were determined by comparing the hypokalemia group with the non‐hypokalemia group for each parameter.

**Results:**

This study included 304 patients who received yokukansan treatment between April 2009 and March 2019. We found that 17.4% (*n* = 53) of the patients experienced yokukansan‐induced hypokalemia. Risk factors detected as significantly different between patients with and without yokukansan‐associated hypokalemia were low serum potassium concentration before yokukansan administration, dose 7.5 g /day or more, and dementia. Hypokalemia occurred earlier in patients with low albumin, low potassium, and dementia.

**Conclusion:**

It is necessary to pay attention to hypokalemia onset when administering yokukansan at 7.5 g or more to patients with low potassium levels and dementia. Our findings suggest that potassium levels must be checked early after yokukansan administration, especially in patients with low albumin, low potassium, and dementia.

## Graphical abstract

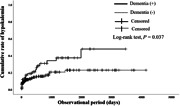

## INTRODUCTION

Yokukansan, a traditional Japanese herbal medicine approved in Japan, consists of seven crude drugs (*Atractylodes lancea* rhizome, *Poria sclerotium*, *Cnidium* rhizome, *Uncaria* hook, Japanese *Angelica* root, *Bupleurum* root, and *Glycyrrhiza*). Glycyrrhizic acid, present in *Glycyrrhiza*, may cause pseudoaldosteronism, consequently hypokalemia.^1^ The compounds responsible for pseudoaldosteronism have not yet been identified, although glycyrrhetinic acid, 3‐monoglucuronyl‐glycyrrhetinic acid, 18*β*‐glycyrrhetyl‐3‐*O*‐sulfate, and other compounds metabolized and produced from glycyrrhizic acid are considered possible causative compounds.^2^, ^3^, ^4^ Glycyrrhizic acid metabolites inhibit 11β‐hydroxysteroid dehydrogenase type 2, an enzyme that catalyzes the conversion of cortisol to cortisone, which in turn raises cortisol levels in tissues. As a result, cortisol over‐binds to mineralocorticoid receptors.^5^, ^6^ Activation of mineralocorticoid receptors increases sodium reuptake, inhibits renal potassium reuptake, and causes hypokalemia.^7^

Yokukansan was initially used for hypersensitivity and night crying in children. It has also been used in a wide range of age groups to treat various mental symptoms. In particular, its use for behavioral and psychological symptoms of dementia (BPSD) is increasing,^8^, ^9^, ^10^, ^11^, ^12^, ^13^, ^14^, ^15^ therefore the risk factors for hypokalemia due to yokukansan use may have changed.

Although several risk factors have been reported for the development of hypokalemia due to yokukansan administration, the risk factors for each psychiatric disorder have not been fully investigated. Detailed patient information may not be available in psychiatry because some patients do not allow blood tests. It is therefore useful to establish the risk of hypokalemia due to yokukansan for each psychiatric disorder. If we could predict which patients are likely to develop hypokalemia before yokukansan administration, we would be able to take measures to prevent it from developing or worsening. We therefore conducted a retrospective cohort study to comprehensively assess the risk of hypokalemia due to yokukansan use for psychiatric disorders.

## METHODS

### Patients

We retrospectively investigated patients who underwent treatment for psychiatric disorders at the Department of Psychiatry, Miyazaki University Hospital, and received yokukansan as a treatment for BPSD or other psychiatric disorders. Eight hundred forty‐six patients received yokukansan at the University of Miyazaki Hospital from April 2009 to March 2019. Some patients were excluded because serum potassium levels were not measured before yokukansan administration (135 patients) or during yokukansan administration (280 patients). Patients who took yokukansan before treatment (92 patients) or before the investigation period (21 patients) were also excluded. To minimize the effects of physical illness, patients with serum potassium levels <3.6 mmol/l who had already developed hypokalemia before the administration of yokukansan (14 patients) were excluded.

Consequently, 304 patients were included in the analysis. The study period was from the start of yokukansan administration to either the discontinuation date or the last traceable date of the yokukansan treatment (December 2020). Hypokalemia was defined using Common Terminology Criteria for Adverse Events (CTCAE) v.5. Patients who had a serum potassium concentration of <3.6 mmol/l (Grade 1 or higher) after yokukansan administration were included in the hypokalemia development group.

Factors affecting the hypokalemia onset, including sex, age, total protein (TP; g/dl), total bilirubin (TB; mg/dl), direct bilirubin (DB; mg/dl), albumin (ALB; g/dl), serum creatinine (S‐Cr; mg/dl), serum sodium, serum potassium, aspartate aminotransferase (AST; U/l), and alanine aminotransferase (ALT; U/l) levels, and the yokukansan dose (maximum dose during the observation period) were investigated. We also investigated the presence or absence of an F00–F98 diagnosis in the International Classification of Diseases, 10th edition (ICD‐10) to compare the incidence of hypokalemia in patients with and without a psychiatric diagnosis. If the psychiatric disorder diagnosed with G00–G99 of ICD‐10 was the same, it was extracted by replacing it with F00–F98. We investigated the presence or absence of the co‐administration of diuretics and corticosteroids, and compared the incidences of hypokalemia.

### Statistical analysis

For continuous data, the unpaired *t*‐test was used to compare the mean values between the two groups, and the Mann–Whitney *U* test was used to compare the median values between the two groups. We used the *χ* ^2^or Fisher's exact test for the nominal variable data. To analyze the odds ratios (ORs) of risk factors for the development of hypokalemia, multiple logistic regression analysis was conducted. Kaplan–Meier curves with and without risk factors were compared using the log‐rank test to analyze the time of hypokalemia onset. In the hypokalemia group, the period from the start of yokukansan to the onset of hypokalemia was plotted. In the nonhypokalemia group, the period from the start to the end of yokukansan administration at the hospital was plotted as censoring. Statistical analysis was conducted using SPSS version 23 for Windows (IBM), and the significance level was set to <5%.

## RESULTS

We included 304 patients in this study, and the median (range) period of yokukansan administration was 136.5 days (range 4–3591 days). The onset of hypokalemia was observed in 53 cases (17.4%: Grades 1–2, 50 cases; Grade 3, two cases; Grade 4, one case), and the median (range) onset time was 42 days (2–1981 days).

A significant difference was found in the median TP (*P* = 0.039) and potassium (*P* = 0.016) levels between patients with and without hypokalemia before yokukansan administration. Analysis of categorized factors showed significant differences in potassium levels of 4.0 mmol/l or less (*P* = 0.037), with a dose of 7.5 g/day or more (*P* = 0.025), and F00–F09 (*P* = 0.004) (Table 1).

**Table 1:** Comparisons of each risk factor in the yokukansan‐treated group with and without development of hypokalemia.

	Hypokalemia (−)	Hypokalemia (+)	*P* value
Number of patients	251	53	
Sex			
Men, *n* (%)	104 (41.4)	21 (39.6)	0.808^a^
Age, years			
Mean ± SD	60.2 ± 20.3	62.2 ± 20.6	0.508^b^
Median (range)	66 (4–88)	69 (16–91)	0.427^c^
≥70 years, *n* (%)	100 (39.8)	28 (52.8)	0.082^a^
TP, g/dl			
Mean ± SD	6.79 ± 0.65	6.59 ± 0.62	0.047* ^,^ b
Median (range)	6.86 (4.63–8.30)	6.68 (5.00–7.67)	0.039* ^,^ c
TP < 6.5 g/dl, *n* (%)**	82 (32.8)	20 (37.7)	0.490^a^
TB, mg/dl			
Mean ± SD	0.62 ± 0.29	0.67 ± 0.37	0.248^b^
Median (range)	0.60 (0.20–2.00)	0.60 (0.30–2.30)	0.396^c^
TB > 1.2 mg/dl, *n* (%)**	12 (4.8)	3 (5.7)	0.732^d^
DB, mg/dl			
Mean ± SD	0.03 ± 0.17	0.07 ± 0.26	0.320^b^
Median (range)	0.00 (0.00–1.00)	0.00 (0.00–1.00)	－
DB > 0.4 mg/dl, *n* (%)****	6 (2.9)	3 (7.1)	0.183^d^
ALB, g/dl			
Mean ± SD	3.84 ± 0.55	3.77 ± 0.56	0.407^b^
Median (range)	3.94 (1.99–4.95)	3.79 (2.51–4.89)	0.321^c^
ALB < 3.9 g/dl, *n* (%)***	114 (45.8)	31 (58.6)	0.069^a^
S‐Cr, mg/dl			
Mean ± SD	0.81 ± 0.43	0.85 ± 0.78	0.554^b^
Median (range)	0.73 (0.21–4.84)	0.71 (0.33–6.17)	0.563^c^
S‐Cr ≥ 0.8 (women), ≥1.05 (men) mg/dl**	52 (20.8)	9 (17.0)	0.529^a^
Sodium, mmol/l			
Mean ± SD	139.5 ± 3.27	140.0 ± 4.17	0.370^b^
Median (range)	140 (125–148)	141 (125–146)	0.078^c^
Sodium < 137 mmol/l, *n* (%)	31 (12.4)	5 (9.40)	0.647^d^
Potassium, mmol/l			
Mean ± SD	4.2 ± 0.39	4.10 ± 0.39	0.061^b^
Median (range)	4.2 (3.6–5.7)	4.0 (3.6–5.3)	0.016* ^,^ c
Potassium ≤ 4.0 mmol/l, *n* (%)	68 (27.1)	22 (41.5)	0.037* ^,^ a
AST, mmol/l			
Mean ± SD	23.0 ± 12.3	24.0 ± 11.0	0.591^b^
Median (range)	20 (9–131)	22 (11–68)	0.333^c^
AST > 38.0 U/l, *n* (%)	15 (6.0)	4 (7.5)	0.754^d^
ALT, mmol/l			
Mean ± SD	8.6 ± 16.1	11.4 ± 14.0	0.247^b^
Median (range)	0 (0–125)	10 (0–65)	－
ALT > 44.0 U/l, *n* (%)	22 (8.8)	4 (7.5)	1.000^d^
Dose of yokukansan, g/day			
Mean ± SD	6.03 ± 2.03	6.43 ± 2.25	0.219^b^
Median (range)	7.50 (1.25–15.0)	7.50 (2.50–15.0)	0.173^c^
Dose ≥ 7.5 g/day	133 (53.0)	37 (69.8)	0.025* ^,^ a
ICD‐10			
F00–F09, *n* (%)	102 (40.6)	33 (62.3)	0.004* ^,^ a
F10–F19, *n* (%)	11 (4.4)	2 (3.8)	1.000^d^
F20–F29, *n* (%)	120 (47.8)	27 (50.9)	0.678^a^
F30–F39, *n* (%)	131 (52.2)	24 (45.3)	0.361^a^
F40–F48, *n* (%)	132 (52.6)	27 (50.9)	0.827^a^
F50–F59, *n* (%)	9 (3.6)	3 (5.7)	0.445^d^
F60–F69, *n* (%)	5 (2.0)	1 (1.9)	1.000^d^
F70–F79, *n* (%)	15 (6.0)	2 (3.8)	0.746^d^
F80–F89, *n* (%)	8 (3.2)	0 (0.0)	－
F90–F98, *n* (%)	3 (1.2)	0 (0.0)	－
Co‐administration			
Diuretics, *n* (%)	13 (5.2)	3 (5.7)	1.000^d^
Corticoids, *n* (%)	17 (6.8)	4 (7.5)	0.770^d^

*Note*: Data are presented as number of patients (mean ± SD) or median (range).

Abbreviations: ALB, albumin; ALT, alanine aminotransferase; AST, aspartate aminotransferase; DB, direct bilirubin; S‐Cr, serum creatinine; SD, standard deviation; TB, total bilirubin; TP, total protein.

F00–F09, organic, including symptomatic mental disorders.

F10–F19, mental and behavioral disorders due to psychoactive substance use.

F20–F29, schizophrenia, schizotypal, and delusional disorders.

F30–F39, mood [affective] disorders.

F40–F48, neurotic, stress‐related, and somatoform disorders.

F50–F59, behavioral syndromes associated with physiological disturbances and physical factors.

F60–F69, disorders of adult personality and behavior.

F70–F79, mental retardation.

F80–F89, disorders of psychological development.

F90–F98, behavioral and emotional disorders with onset usually occurring in childhood and adolescence.

^a^ *χ* ^2^ test.

^b^ Unpaired *t*‐test.

^c^ Mann–Whitney *U* test.

^d^ Fisher exact test.

* *P* < 0.05.

** One missing value.

*** Three missing values.

**** 57 missing values.

Shimada et al. reported that a potassium level of ≤4.0 mmol/l is a risk factor for developing hypokalemia due to yokukansan.^16^ The present study excluded patients with serum potassium levels <3.6 mmol/l before yokukansan administration, therefore the group with a serum potassium concentration of 3.6–4.0 mmol/l and the group with a serum potassium concentration exceeding 4.0 mmol/l were compared. On the basis of these results, we conducted a logistic regression analysis. Since there was a significant difference in the F00–F09 group, we analyzed dementia (56.3%), which was most abundant in the F00–F09 group in this study. In the logistic regression analysis, potassium levels of ≤4.0 mmol/l (OR = 2.073, 95% confidence interval [CI] = 1.100–3.908, *P* = 0.024), 7.5 g of yokukansan or higher (OR = 2.001, 95% CI = 1.040–3.851, *P* = 0.038), and dementia (OR = 2.294, 95% CI = 1.203–4.373, *P* = 0.012) were identified as risk factors (Table 2).

**Table 2:** Regression analysis using hypokalemia as dependent variable in yokukansan‐treated group.

	Odds ratio	95% CI	*P* value
TP (g/dl)**	0.643	0.402–1.027	0.065
Potassium (mmol/l)	0.45	0.174–1.161	0.099
Potassium ≤4.0 mmol/l	2.073	1.100–3.908	0.024*
Dose ≥7.5 g/day	2.001	1.040–3.851	0.038*
Dementia	2.294	1.203–4.373	0.012*

Abbreviation: CI, confidence interval.

* *P* < 0.05.

** One missing value.

**Figure 1:**
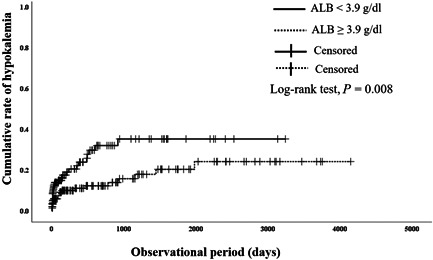
Effects of low albumin (ALB) (<3.9 g/dl) on cumulative rate of hypokalemia after administration of yokukansan. Solid line: patients with low ALB; dotted line: patients without low ALB. Significant difference was observed between patients with and without low ALB in log‐rank test (*P* = 0.008).

**Figure 2:**
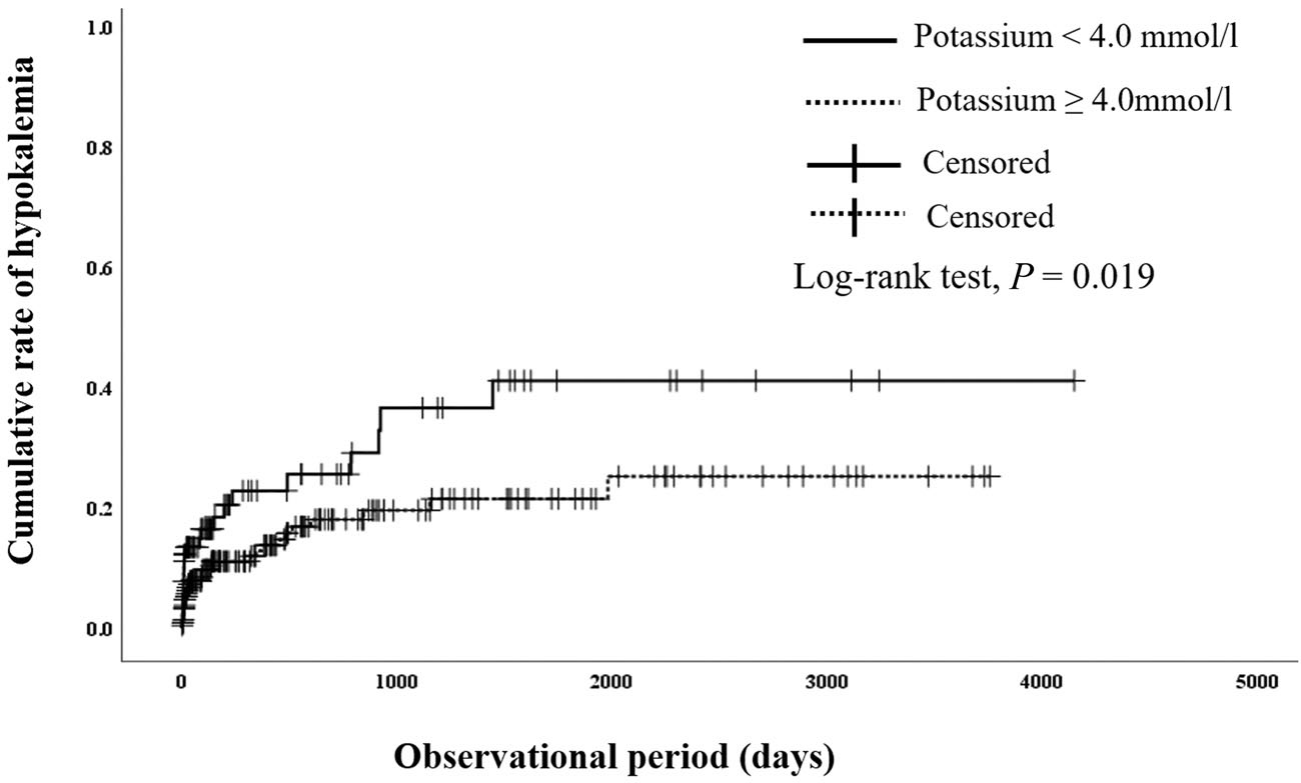
Effects of low potassium (<4.0 mmol/l) on cumulative rate of hypokalemia after administration of yokukansan. Solid line: patients with low potassium; dotted line: patients without low potassium. Significant difference was observed between patients with and without low potassium in log‐rank test (*P* = 0.019).

**Figure 3:**
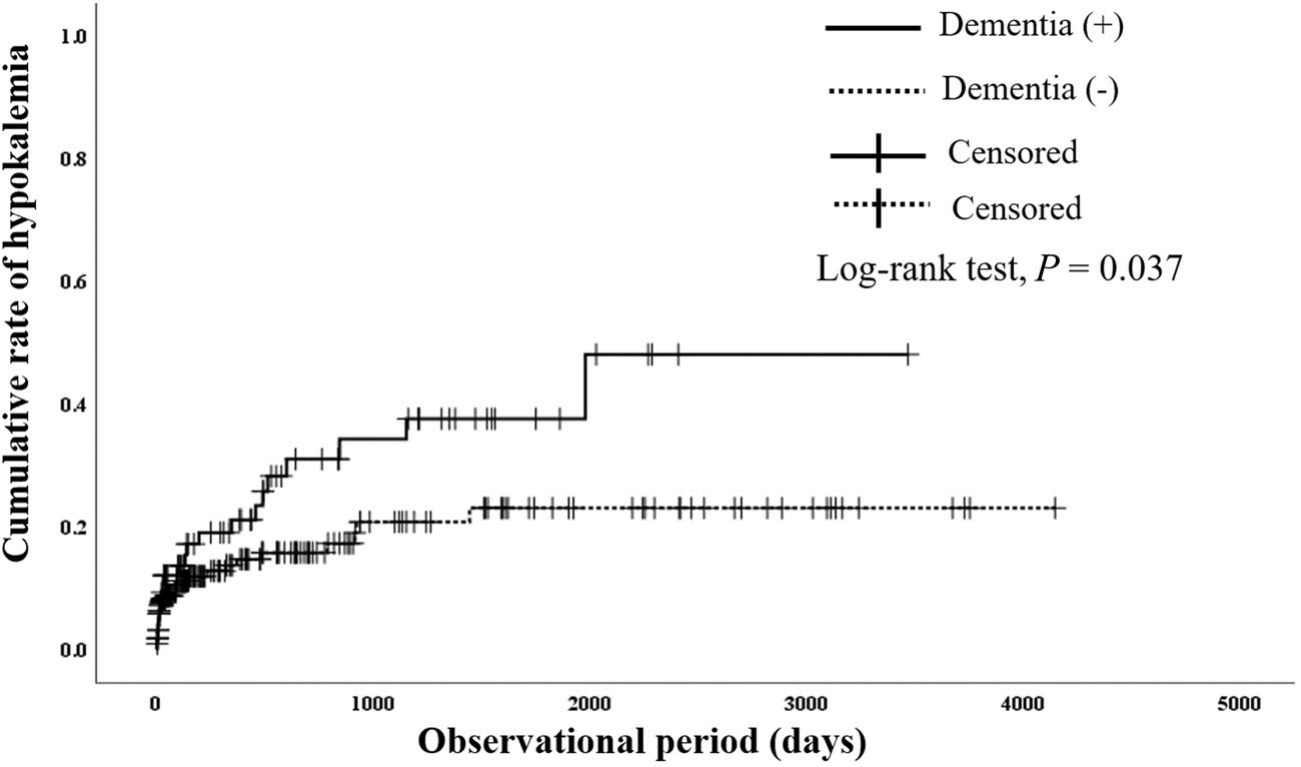
Effects of dementia on cumulative rate of hypokalemia after administration of yokukansan. Solid line: patients with dementia; dotted line: patients without dementia. Significant difference was observed between patients with and without dementia in log‐rank test (*P* = 0.037).

A comparative study was also conducted during the onset of hypokalemia. The time between yokukansan administration and the onset of hypokalemia was compared with and without risk factors to evaluate the risk factors for early onset of hypokalemia. The group with serum albumin <3.9 g/dl or serum potassium <4.0 mmol/l before yokukansan administration had a shorter time of hypokalemia onset than the group with serum albumin ≥3.9 g/dl (*P* = 0.008) (Figure 1) or serum potassium ≥4.0 mmol/l (*P* = 0.019) (Figure 2). Furthermore, the dementia group had a shorter time of hypokalemia onset before yokukansan administration than the nondementia group (*P* = 0.037) (Figure 3). We compared the two groups in accordance with the risk factors of nominal variable data listed in Table 1, but no significant differences were found except for serum albumin <3.9 g/dl, serum potassium <4.0 mmol/l, and dementia (data not shown).

## DISCUSSION

While yokukansan is used to treat various psychiatric symptoms, the hypokalemia associated with the side effects of pseudoaldosteronism impedes the continuation of yokukansan administration, therefore avoiding the onset and aggravation of hypokalemia caused by yokukansan is desirable. This study showed that patients with dementia could be at risk of developing hypokalemia due to yokukansan administration. The hypokalemia incidence in this study was 17.4%. Previous studies reported an incidence of 26.6%, which is similar despite the different patient backgrounds.^16^ Since the median hypokalemia onset time due to yokukansan was 42 days after the start of treatment, it may be necessary to monitor serum potassium levels approximately 1 month after yokukansan administration. In the present study, three risk factors (potassium level of 3.6–4.0 mmol/l, yokukansan does of 7.5 g or higher, and dementia) were identified for hypokalemia onset after yokukansan administration.

The yokukansan dose as one of the risk factors is consistent with that in previous reports. The pseudoaldosteronism associated with yokukansan has been reported to be dose‐dependent.^17^ In particular, caution is required when administering yokukansan at 7.5 g/day, and it is necessary to limit administration to <7.5 g in high‐risk patients.

The present study suggests that patients with low potassium levels (3.6–4.0 mmol/l: not hypokalemia, but relatively low serum potassium levels within the normal range) before yokukansan administration were more likely to develop hypokalemia after yokukansan administration. Previous studies also reported that patients with low potassium levels before yokukansan administration were more likely to develop hypokalemia after yokukansan administration.^16^ In the present study, hypokalemia cases before yokukansan administration were excluded. However, even if the serum potassium concentration was within the normal range, hypokalemia due to yokukansan was more likely to occur in the group with a serum potassium concentration of ≤4.0 mmol/l.

The proportion of patients with dementia was significantly higher in the hypokalemia group. The ICD‐10 classification was used for comparison with other psychiatric disorders. Nonetheless, there were no significant differences, except for F00–F09 and dementia. Hirai et al. reported that hypokalemia due to yokukansan was less likely to occur in patients with dementia.^18^ The reasons for this discrepancy are not clear, but since each patient was analyzed at a single institution, we assume that the differences in patient populations may have caused the discrepancy. A study by Ishida et al. using the Japanese Adverse Drug Event Report database (JADER) reported that patients with dementia were at risk of pseudoaldosteronism due to yokukansan,^19^ which is consistent with the results of the present study. It has been reported that 85.8% of older people with severe dementia admitted to a long‐term care facilities had dietary problems.^20^ Patients with dementia had a background of poor nutritional intake, such as anorexia and dysphagia, which may have contributed to the hypokalemia due to yokukansan administration. Because it is difficult for patients with dementia to be aware of the initial symptoms of pseudoaldosteronism, it is possible that its detection was delayed and the incidence of hypokalemia increased.^19^, ^21^, ^22^

The Kaplan–Meier curve analysis was conducted to compare and identify the factors that accelerated the hypokalemia onset. The group with low albumin (<3.9 g/dl) developed hypokalemia earlier than that of the group with high albumin (≥3.9 g/dl). Since the group with low albumin was undernourished, it is suggested that low potassium levels occurred earlier due to poor nutritional intake. Hypokalemia due to yokukansan appeared earlier in the dementia group than in the nondementia group. It was suggested that patients with low albumin, low potassium, and dementia developed hypokalemia earlier with yokukansan administration. We suggest that patients with dementia not only have a risk of developing hypokalemia due to yokukansan but also have a risk of developing hypokalemia earlier. When using yokukansan in patients with dementia, it is necessary to pay close attention to hypokalemia.

We found that yokukansan (7.5 g/day), low serum potassium level (3.6–4.0 mmol/l), and the presence of dementia may be risk factors for hypokalemia onset after the administration of yokukansan. Patients with low serum albumin levels, low serum potassium levels, and dementia before yokukansan may develop hypokalemia early.

One limitation of this study is that it was a single‐center study with a limited number of cases. It was also a retrospective study, and the possibility of selection and information bias cannot be ruled out. We intend to include more cases and conduct a multicenter prospective study. However, this study provides clinically valuable information on the risk factors of hypokalemia development due to yokukansan administration.

## CONCLUSION

This study showed that patients with low potassium levels before administering yokukansan at doses of 7.5 g or more had a higher incidence of hypokalemia. It also showed that patients with low serum albumin, low serum potassium, and dementia developed hypokalemia earlier. Considering these findings, we recommend monitoring serum potassium levels frequently in patients with multiple risk factors.

## AUTHOR CONTRIBUTIONS

All the listed authors meet the authorship criteria and agree with the manuscript's content.

## CONFLICT OF INTEREST STATEMENT

The authors declare no conflict of interest.

## ETHICS APPROVAL STATEMENT

This study was conducted with the approval of the Ethics Review Committee of the University of Miyazaki Hospital per the ethical guidelines for medical research for humans, and the protocol complied with the Declaration of Helsinki.

## CLINICAL TRIAL REGISTRATION

Committee of the Faculty of Medicine, University of Miyazaki, Approval No. O‐0579.

## PATIENT CONSENT STATEMENT

The requirement for informed consent was waived due to the study's retrospective nature. The details of the study are described on a web page that patients can access from the hospital's website. If the patients do not want to attend the study, they can inform us of their intentions.

## Data Availability

We cannot open the raw data to the public. The disclosure of personal data was not planned in the research protocol approved by the institutional review board.
